# Repetitive Transcranial Magnetic Stimulation for Neuropathic Pain and Neuropsychiatric Symptoms in Traumatic Brain Injury: A Systematic Review and Meta-Analysis

**DOI:** 10.1155/2022/2036736

**Published:** 2022-07-30

**Authors:** Xin Li, Tijiang Lu, Hong Yu, Jie Shen, Zhengquan Chen, Xiaoyan Yang, Zefan Huang, Yuqi Yang, Yufei Feng, Xuan Zhou, Qing Du

**Affiliations:** ^1^Department of Rehabilitation, Xinhua Hospital, Shanghai Jiao Tong University School of Medicine, Shanghai 200092, China; ^2^School of Kinesiology, Shanghai University of Sport, Shanghai 200438, China; ^3^Rehabilitation Medical Center, Jiaxing Second Hospital, Jiaxing, Zhejiang 314000, China; ^4^College of Global Public Health, New York University, New York, NY 10003, USA; ^5^Chongming Branch of Xinhua Hospital, School of Medicine, Shanghai Jiao Tong University, Shanghai 202150, China

## Abstract

Neuropathic pain and neuropsychiatric symptoms are common complications reported by the traumatic brain injury (TBI) population. Although a growing body of research has indicated the effectiveness of repetitive transcranial magnetic stimulation (rTMS) for the management of neurological and psychiatric disorders, little evidence has been presented to support the effects of rTMS on neuropathic pain and neuropsychiatric symptoms in patients with TBI in all age groups. In addition, a better understanding of the potential factors that might influence the therapeutic effect of rTMS is necessary. The objective of this preregistered systematic review and meta-analysis was to quantify the effects of rTMS on physical and psychological symptoms in individuals with TBI. We systematically searched six databases for randomized controlled trials (RCTs) of rTMS in TBI patients reporting pain and neuropsychiatric outcomes published until March 20, 2022. The mean difference (MD) with 95% confidence intervals (CIs) was estimated separately for outcomes to understand the mean effect size. Twelve RCTs with 276 TBI patients were ultimately selected from 1605 records for systematic review, and 11 of the studies were included in the meta-analysis. Overall, five of the included studies showed a low risk of bias. The effects of rTMS on neuropathic pain were statistically significant (MD = −1.00, 95% CI -1.76 to -0.25, *P* = 0.009), with high heterogeneity (*I*^2^ = 76%). A significant advantage of 1 Hz rTMS over the right dorsolateral prefrontal cortex (DLPFC) in improving depression (MD = −6.52, 95% CI -11.58 to -1.46, *P* = 0.01) was shown, and a significant improvement was noted in the Rivermead Post-Concussion Symptoms Questionnaire-13 (RPQ-13) scores of mild TBI patients after rTMS (MD = −5.87, 95% CI -10.63 to -1.11, *P* = 0.02). However, no significance was found in cognition measurement. No major adverse events related to rTMS were reported. Moderate evidence suggests that rTMS can effectively and safely improve neuropathic pain, while its effectiveness on depression, postconcussion symptoms, and cognition is limited. More trials with a larger number of participants are needed to draw firm conclusions. This trial is registered with PROSPERO (PROSPERO registration number: CRD42021242364.

## 1. Introduction

Traumatic brain injury (TBI) is caused by a violent bump, blow, or jolt to the head or a penetrating head injury with substantial neurological disabilities and mental distress. It remains a global health problem, with an annual incidence of 200-1967 cases/100,000 individuals [1]. Approximately half of TBI patients do not reach the preinjury functional level within 1 year, and more than 50% of moderate/severe TBI patients are unable to return to work at 2 and 5 years postinjury [2, 3], which presents a substantial economic burden to victims, their families, and society. Some promising noninvasive-based approaches have emerged to relieve pain and to improve neural connectivity in people with TBI. Transcranial magnetic stimulation (TMS) is a noninvasive, painless interventional method that induces nerve cell activity in superficial areas of the sensory-motor circuits and facilitates plastic changes in neural networks [4, 5]. Repeated application of TMS at regular intervals, also called repetitive TMS (rTMS), is a tool to enhance clinical recovery in both mild [6, 7] and more severe TBI patients [8–10]. rTMS treatment acts through an electromagnetic field created by a coil placed on the scalp [11], generating a superficial cortical current that is capable of changing neuron activity, even in brain regions that are distant from the stimulation site.

The categorization of TBI into severe, moderate, and mild by scores on the Glasgow coma scale (GCS) is based on clinical grounds (including responses assessed in the visual, motor, and verbal domains) and standard brain imaging. Patients with mild TBI have GCS scores of 13–15 with full neurological recovery, those with moderate TBI have GCS scores of 9–12 with a decreased level of consciousness, and those with severe TBI have GCS scores of 3–8 with coma [12]. Corrigan and Hammond reported that nearly 60% of patients surviving moderate to severe TBI complained of cognitive deficits and behavioral changes [13], which present major barriers to positive social outcomes, such as community reintegration and employment, among post-TBI patients and generate a major socioeconomic impact [14].

Neuropathic pain is another common complication reported by 57.8% of the TBI population, and the cumulative incidence of headache was almost 91% 1 year after mild TBI [15, 16]. Moreover, central pain, which is caused by a lesion or dysfunction of the somatosensory nervous system within the central nervous system and presents as neuropathic pain (such as headache) [17], has been reported to have a similar prevalence across brain trauma severity levels, potentially making it the most prevalent form of chronic pain associated with moderate-to-severe TBI [18]. Patients with central pain usually experience sensations of tingling, chills, itching, and numbness, in addition to pain, as well as abnormal sensations that feel like electrical shocks or burns, especially when numb areas are touched [19], leading to limited functional recovery, impairment in activities of daily living, and poor quality of life.

Previous brain stimulation techniques, including rTMS, were recommended in cognitive rehabilitation, with working memory seeming particularly amenable to enhancement [20]. Studies have shown that low-frequency rTMS at 1 Hz decreases cortical excitability, whereas high-frequency rTMS at ≥5 Hz increases the excitability of the cerebral cortex [21, 22]. More specifically, dorsolateral prefrontal cortex (DLPFC) stimulation has been related to improvements in trauma-related conditions [23], such as neurobehavioral gains, cognitive enhancement, and depression reduction [24, 25].

In addition, rTMS has been recommended by the International Federation of Clinical Neurophysiology for the management of neurological and psychiatric disorders [26], and evidence is now quickly increasing, highlighting that DLPFC-rTMS should relieve pain in patients with chronic pain conditions, including migraine [27], spinal cord injury [26], and fibromyalgia syndrome [28]. Actually, a recent meta-analysis found that high-frequency DLPFC stimulation is able to induce an analgesic effect in patients with chronic pain [29], but at present, the overwhelming majority of systematic reviews and meta-analyses on health-related consequences after TBI have focused on depression, memory, selective attention, and postconcussion syndrome [30–32], with far less attention given to neuropathic pain. Therefore, the present systematic review and meta-analysis was aimed at examining the evidence supporting the effectiveness of an rTMS intervention program for neuropathic pain and neuropsychiatric measurements of patients with TBI.

## 2. Methods

### 2.1. Study Design

This systematic review and meta-analysis was conducted in accordance with the Preferred Reporting Items for Systematic Reviews and Meta-Analysis (PRISMA) guidelines, and the protocol was registered in the PROSPERO database (No. CRD42021242364).

### 2.2. Search Strategy

A comprehensive search was conducted in the PubMed, Embase, Cochrane Library, Cumulative Index of Nursing and Allied Health Literature (CINAHL), and Web of Science databases until March 20, 2022. Key terms were used, including “traumatic brain injury,” “TBI,” “posttraumatic stress disorder,” “PTSD,” “transcranial magnetic stimulation,” and “TMS,” to identify articles on the effect of rTMS on TBI. The search strategies are shown in Appendix S1.

Two reviewers (Xin Li and Xiaoyan Yang) independently assessed the eligibility of the literature. The preliminary screening was based on the titles and abstracts. The selected articles were then evaluated in their entirety. If there was a disagreement, the full text of the article was checked and discussed, if necessary, with third-party adjudication (Yuqi Yang).

### 2.3. Eligibility Criteria and Selection Process

Studies were considered eligible if they met the following criteria: (1) *population*: patients who were diagnosed with TBI, with no restrictions on sex, age, or ethnicity; (2) *intervention*: rTMS; medication was allowed during rTMS; (3) *comparisons*: sham stimulation or any conventional TBI treatment (e.g., pharmacological therapy or nonpharmacological therapy); (4) *outcomes*: neuropathic pain (including central pain and headache) and neuropsychiatric symptoms (including postconcussive symptoms, depression, or cognitive function); and (5) *study design*: randomized controlled trials (RCTs) published in peer-reviewed English journals.

### 2.4. Outcome Measurements and Data Extraction

Change in neuropathic pain was the primary outcome for extraction. Self-reported neuropathic pain was assessed by the numeric pain rating scale (NPRS), an 11-point scale with scores ranging from 0 to 10, where “0” indicates no pain and “10” suggests the most severe pain imaginable. When reported, changes in depression severity evaluated by the Montgomery-Asberg Depression Rating Scale (MADRS), Hamilton Rating Scale for Depression (HRSD), Inventory of Depressive Symptomatology (IDS), or Patient Health Questionnaire-9 (PHQ-9) were extracted, with high scores representing severe depression. Data on postconcussive symptoms and cognition were also extracted as the secondary outcomes, and an MD was calculated from pre- to postintervention.

Two reviewers (Hong Yu and Xuan Zhou) independently extracted data from the included studies. If the opinions were inconsistent, a third reviewer reevaluated the articles and discussed them with the two reviewers to reach an agreement. They extracted the following data using a data extraction form: study design, number of participants, age, duration since TBI/concussion, severity of TBI, medication used, outcome assessed, interventions, comparators, relevant statistical data, and adverse events. The intervention protocols of the rTMS group and control group were extracted and included the following details: coil, intensity, frequency, stimulation pulse/train, target brain region, sessions, study duration, and adverse events.

The mean, standard deviation (SD), and sample size were extracted for the outcome measures in each group (i.e., active and sham) for the pooled analysis. Published protocols were referenced, and the corresponding authors were contacted for additional data when data were not directly available in the article.

### 2.5. Risk of Bias Assessment

Reviewers (Tijiang Lu, Jie Shen, and Zefan Huang) independently assessed the methodological quality of the included studies using the revised Cochrane risk-of-bias assessment tool (RoB 2.0) for RCTs [33]. There are five domains in RoB 2.0: the randomization process, deviations from the intended intervention, missing outcome data, the measurement of the outcome, and selection of the reported outcomes. For missing outcome data in individual studies, we stipulated a low risk of bias for a loss to follow-up of less than 10% and a difference of less than 5% in missing data between intervention and control groups. Publication bias was assessed through visual inspection of funnel plots for each outcome in which 10 or more eligible studies were identified.

### 2.6. Meta-Analysis and Subgroup Analyses

Review Manager Software version 5.3 (Cochrane Collaboration, Oxford, England) was used to analyze the data in this meta-analysis (Xin Li and Qing Du). The effect of rTMS was expressed as the mean difference (MD) with 95% confidence intervals (CIs). The heterogeneity was estimated by using the *I*^2^ test. If the *I*^2^ value was less than 50%, the fixed-effect model was used; otherwise, a random-effect model was used. A statistically significant *P* value was set at 0.05. Moreover, meta-analysis was performed on outcome measures of different postintervention time points according to the included studies. As provoked depression studies evaluated by MADRS reported stimulation over the bilateral, left, or right DLPFC, subgroup analyses were further conducted based on the target brain region.

### 2.7. Certainty of Evidence

We summarized the evidence and assessed its certainty separately for bodies of evidence from RCTs. Two reviewers (Zhengquan Chen and Yufei Feng) used the Grading of Recommendations Assessment, Development and Evaluation (GRADE) methodology to rate the certainty of the evidence for each outcome as high, moderate, low, or very low. Detailed GRADE guidance was used to assess the overall risk of bias, imprecision, inconsistency, indirectness, and publication bias and to summarize the results [34].

## 3. Results

### 3.1. Study Selection

**Figure 1:**
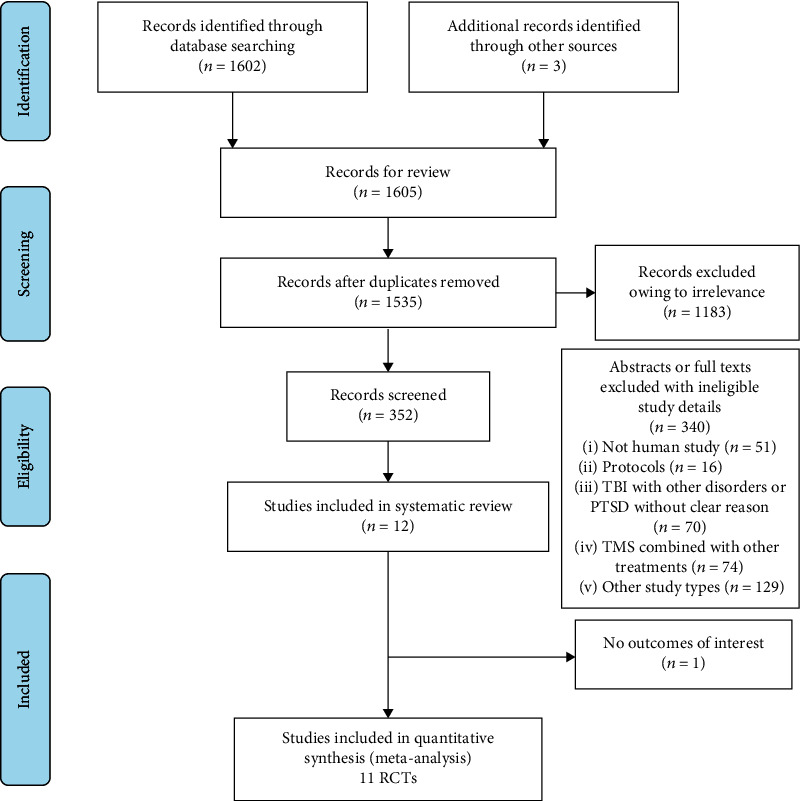
Flow diagram of the selection process.

Twelve RCTs were ultimately selected for systematic review from 1605 records with a total of 276 TBI patients [7, 10, 35–45], and 11 of them with 236 patients were included in the meta-analysis [7, 35–43, 45], as shown in Figure 1.

The baseline demographic and clinical characteristics of the included studies are shown in Table 1. The age of the included patients was between 14 and 65 years. The included studies reported neuropathic pain (including posttraumatic headache), posttraumatic stress disorder (PTSD) symptoms, or mental health issues, such as declines in cognitive function and depression. In most of the studies, the intervention groups were treated with both rTMS and drugs. All patients were in subacute and chronic stages, from 3 weeks to over 20 years after their injuries. The follow-up time was up to 24 weeks after rTMS treatment.

Four included studies stimulated the left DLPFC with 10-20 Hz high-frequency rTMS [35–37, 42], while others applied rTMS over the right DLPFC (*n* = 3) [41, 43, 45], the bilateral DLPFC (*n* = 2) [38, 39], or the motor cortex (*n* = 2) [7, 40] with stimulation of 1 Hz or 10 Hz. Four studies used 70%-90% resting motor threshold (RMT) subthreshold stimulation [7, 35, 40, 42], and 7 studies used 100% RMT to 120% RMT suprathreshold stimulation [36–39, 41, 43, 45]. The intervention duration ranged from 5 days to 4 weeks, with frequencies ranging from 3 sessions per week to 20 sessions per day.  Table 2 summarizes the detailed intervention protocols of the rTMS interventions and sham interventions in the 11 articles.

The risk of bias measured by the RoB 2.0 tool in the 11 studies included for meta-analysis is presented in Table 3. Overall, five studies showed a low risk of bias. Ten RCTs generated an adequately randomized sequence, and eight of them were conducted using a blinded method for the outcome measurement. Ratings using the GRADE methodology for all outcome measurements were inconsistent and ranged from moderate to very low quality (see Appendix S2); therefore, most studies were classified as fair.

### 3.2. Primary Outcome

#### 3.2.1. Neuropathic Pain

**Figure 2:**
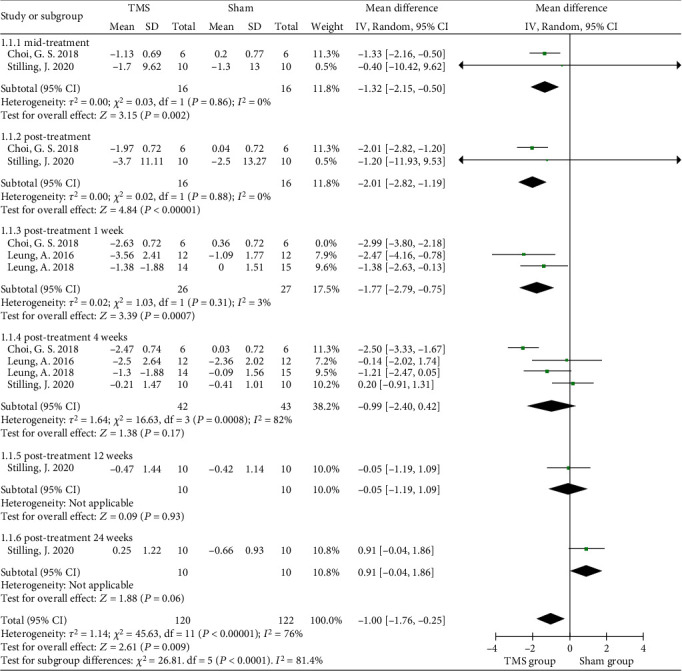
Forest plots of the different-term effects of rTMS on self-reported neuropathic pain in TBI.

Three studies investigated the effect of rTMS on chronic posttraumatic headache (over 3 months) [7, 35, 42], whereas one study included patients with central pain (consisting of shooting pain, burning pain, etc.) lasting for 6 months [40]. In the three studies examining headache, the duration of treatment was from 1 week to 2 weeks (3 sessions to 10 sessions), whereas in the study of central pain, the intervention frequency was 5 sessions per week, lasting for 2 weeks. When the data from four randomized controlled studies were pooled, significant improvement in pain was found to be associated with rTMS in TBI patients (MD = −1.00, 95% CI -1.76 to -0.25, *P* = 0.009). However, there was strong evidence of heterogeneity (*I*^2^ = 76%) (Figure 2), which might be due to the inconsistent distinct targeted brain regions and diversified follow-up durations.

**Figure 3:**
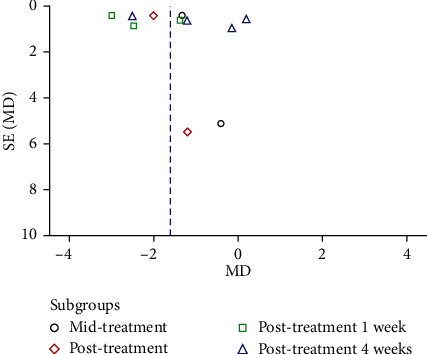
Funnel plot regarding self-reported neuropathic pain in the rTMS group compared with the control group.

The four studies had an intervention duration of 1 to 2 weeks, and the outcome measures were collected at baseline and posttreatment (or 1 week posttreatment), with at least 4 weeks of observation after the intervention. The pooled results showed midtreatment and posttreatment effects, as a significant analgesic effect was found in the rTMS group (MD = −1.32, 95% CI -2.15 to -0.50, *P* = 0.002 and MD = −2.01, 95% CI -2.82 to -1.19, *P* < 0.0001, respectively) [35, 40]. The pooled data of three studies showed that a significant change in pain in the rTMS group was found at the 1-week follow-up after treatment (MD = −1.77, 95% CI -2.79 to -0.75, *P* < 0.001, *I*^2^ = 3%) [7, 40, 42]. After four weeks of follow-up, no significant differences were found between the rTMS and sham control groups (MD = −0.99, 95% CI -2.40 to 0.42, *P* = 0.17) with high heterogeneity (*I*^2^ = 82%), which seemed to be associated with the small number of rTMS sessions reported in the study of Leung et al. [7, 42]. Only Stilling et al. reported the mean changes in headache severity at 3 months and 6 months postintervention, but no significant difference was revealed (*P* = 0.93 and *P* = 0.06, respectively) (Figure 2) [35]. In addition, the funnel plot showed an asymmetrical distribution regarding central pain or headache, suggesting a high risk of publication bias (Figure 3).

### 3.3. Secondary Outcomes

#### 3.3.1. Depression

**Figure 4:**
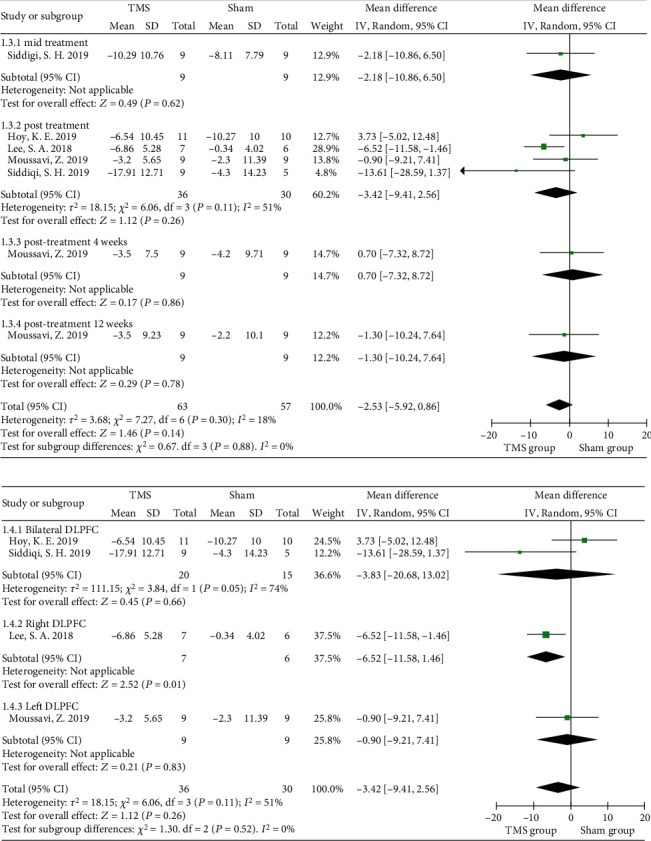
Forest plots of the effect of rTMS on depression measured by the MADRS in TBI patients. (a) Total analysis; (b) subgroup analysis of posttreatment effectiveness. DLPFC: dorsolateral prefrontal cortex; MADRS: Montgomery-Asberg Depression Rating Scale.

**Figure 5:**
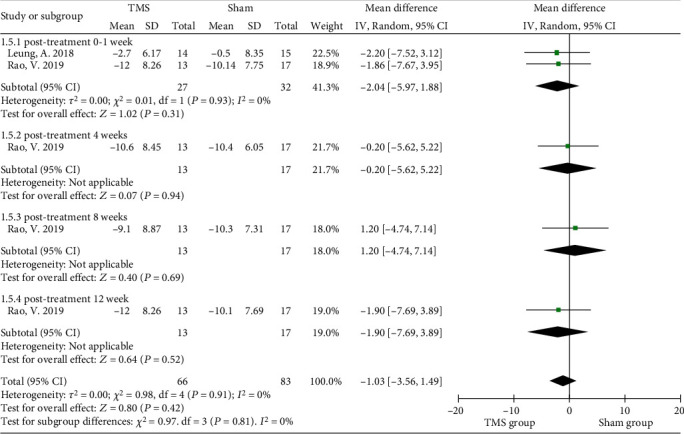
Forest plots of the effect of rTMS on depression measured by the HRSD in TBI patients. HRSD: Hamilton Rating Scale for Depression.

**Figure 6:**
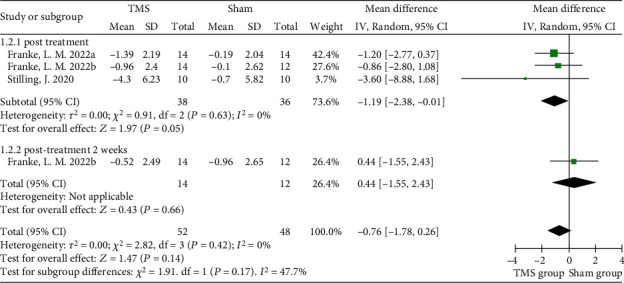
Forest plots of different-term effects of rTMS on depression measured by the PHQ-9 in TBI patients. PHQ-9: Patient Health Questionnaire-9.

Nine of the included studies evaluated the effect of rTMS on depression [7, 35, 36, 38, 39, 41–43, 45], and the Montgomery-Asberg Depression Rating Scale (MADRS), Hamilton Rating Scale for Depression (HRSD), Inventory of Depressive Symptomatology (IDS), or Patient Health Questionnaire-9 (PHQ-9) was used to rate depression, with high scores representing severe depression. The interventional protocol in the nine studies also varied from 3 sessions of 80% RMT intensity to 20 sessions of 120% RMT intensity. No significant improvement in depression was found after rTMS intervention (MD = −2.53, 95% CI -5.92 to 0.86, *P* = 0.14, *I*^2^ = 18%) when the MADRS data from four randomized controlled studies were pooled (Figure 4(a)). Subgroup analysis of MADRS data showed no significant difference between groups of patients receiving either bilateral (*P* = 0.66) or left rTMS (*P* = 0.83) on DLPFC areas immediately posttreatment, while Lee and Kim introduced a significant advantage of 1 Hz right DLPFC-rTMS in improving depression (MD = −6.52, 95% CI -11.58 to -1.46, *P* = 0.01) (Figure 4(b)) [41]. Similarly, no significant improvement in depressive symptoms was found using HRSD scores (MD = −1.03, 95% CI -3.56 to 1.49, *P* = 0.42, *I*^2^ = 0%) (Figure 5). The PHQ-9 was used in 2 studies, and no significant result was found (MD = −0.76, 95% CI -1.78 to 0.26, *P* = 0.14, *I*^2^ = 47.7%) (Figure 6).

#### 3.3.2. Postconcussive Symptoms

**Figure 7:**
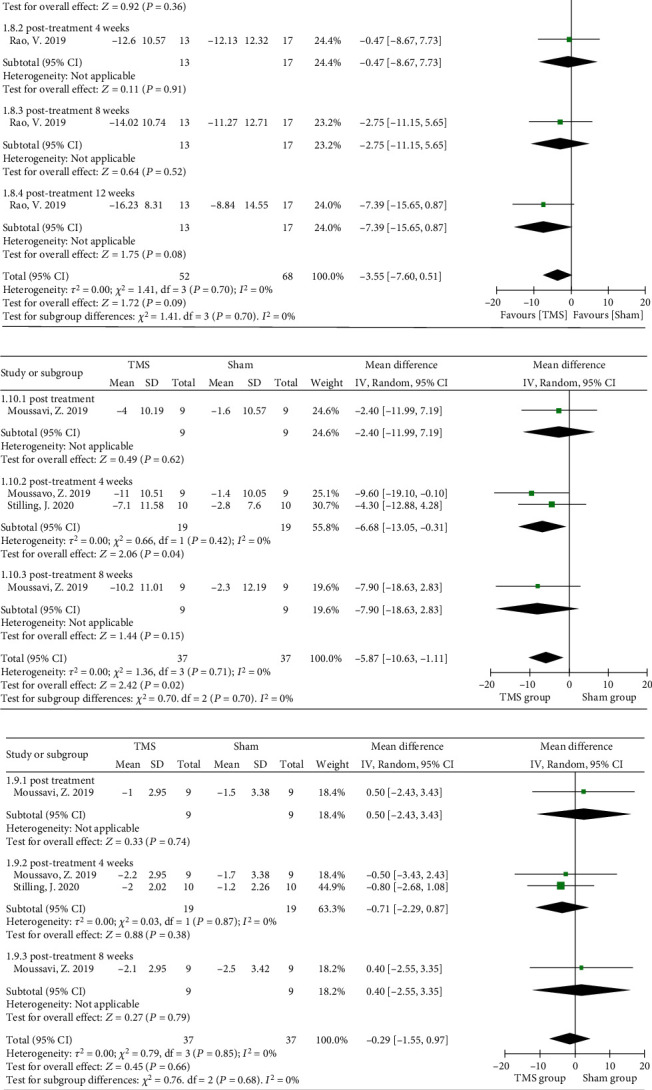
Forest plots of different parts of rTMS on the severity of different symptoms measured by the RPQ in TBI patients: (a) the RPQ; (b) the RPQ-13; (c) the RPQ-3. RPQ: Rivermead Post-Concussion Questionnaire.

The Rivermead Post-Concussion Symptoms Questionnaire (RPQ) is a self-reported and reliable measure of PCS. Scores from the 16 RPQ questions can range from 0 to 64, as symptoms are rated on a 4-point Likert scale, ranging from “not experienced at all” to “a severe problem” [46]. In the meta-analysis, the 16 questions making up the RPQ were only used in one RCT [43], and the pooled data among varied follow-up durations showed no significant difference (MD = −3.55, 95% CI -7.60 to 0.51, *P* = 0.09) (Figure 7(a)). Otherwise, the RPQ could be broken into the RPQ-13 (cognitive and emotional) and the RPQ-3 (headaches, dizziness, and nausea) to form a unidimensional construct [47]. The subgroup analysis showed that rTMS over the left DLPFC could generate a significant and sustained improvement, especially at 4 weeks of follow-up, as measured by the RPQ-13 scores (MD = −5.87, 95% CI -10.63 to -1.11, *P* = 0.02, *I*^2^ = 0%) (Figure 7(b)). However, no significant changes were found in the RPQ-3 scores of mild TBI patients between the rTMS and sham groups after intervention (*P* = 0.66, Figure 7(c)). In addition, postconcussive symptoms were not explored in the included studies that recruited moderate and severe TBI patients.

#### 3.3.3. Cognition

**Figure 8:**
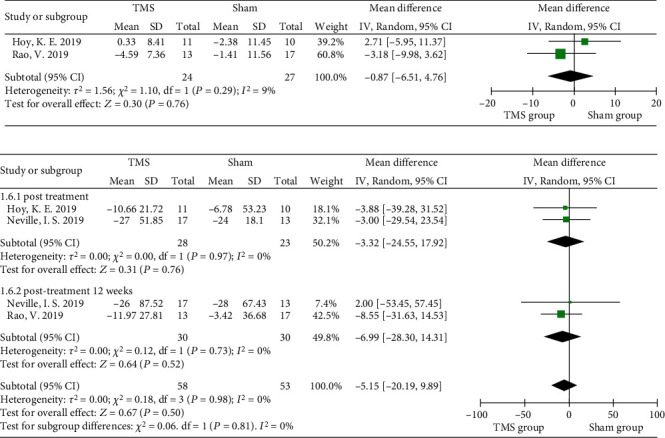
Forest plots of different parts of rTMS on cognition measured by the TMT-A and TMT-B in TBI patients: (a) the TMT-A; (b) the TMT-B. TMT: Trail Making Test.

**Figure 9:**
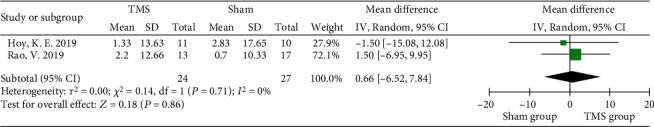
Forest plots of different parts of rTMS on cognition measured by the SCWT in TBI patients. SCWT: Stroop Color-Word Test.

A large variety of questionnaires and cognitive tests were used in the included studies, such as the Wechsler Adult Intelligence Scale (WAIS) and Montreal Cognitive Assessment (MoCA). Due to the insufficient number of studies using the WAIS, data pooling could not be executed. However, the Trailmaking Test (TMT) [37, 38, 43] and Stroop Color-Word Test (SCWT) [38, 41, 43] were used in three included studies. The TMT is a psychological test scoring the time spent connecting numbered circles in sequential order, with the TMT-A and TMT-B representing two subtests by connecting numbered circles in specific order. A meta-analysis revealed insignificant changes in TMT-A (MD = −0.87, 95% CI −6.51 to 4.76, *P* = 0.76, *I*^2^ = 9%) and TMT-B (MD = −5.15, 95% CI −20.19 to 9.89, *P* = 0.5, *I*^2^ = 0%) scores after rTMS intervention (Figure 8). Three RCTs used the SCWT to assess the ability to inhibit cognitive interference; two of the RCTs recorded the number of words (word task), number of bar colors (color task), and number of color words (color-word task) spoken within a specified time, while the other RCT analyzed the accumulated time for completing the 3 tasks [41]. No significant difference was revealed from the pooled analysis (MD = 0.66, 95% CI -6.52 to 7.84, *P* = 0.86, *I*^2^ = 0%) (Figure 9).

#### 3.3.4. Adverse Events

The included RCTs did not report major adverse events, such as vomiting or syncope, during the rTMS interventions, although several mild side effects were reported, including headache, scalp discomfort, toothache, transient twitching, or neck discomfort (Table 2). In one study, the rate of side effects was up to 70.6% during the rTMS intervention.

## 4. Discussion

The aims of the present systematic review and meta-analysis were to clarify the effects of rTMS on neuropathic pain and neuropsychiatric functional measurements in patients with TBI. The results of the meta-analysis including 11 studies indicated that rTMS could induce significant analgesic effects, especially for headaches, although there was large heterogeneity in the rTMS interventional protocols that were followed. Compared to the sham control groups, the rTMS groups showed significant changes in postconcussive symptoms (measured by the RPQ-13). However, rTMS did not seem to improve depression and cognitive function, as the changes did not reach statistical significance.

Although headache or central pain gradually decreased with recovery from TBI, significant improvement in pain was found in the rTMS group. The ability of motor cortex rTMS to interfere with the processing of acute provoked pain was demonstrated by Lefaucheur et al., even if there was underlying chronic neuropathic pain [48]. In the quantitative analysis of neuropathic pain, the stimulated regions, including the left or right DLPFC, the bilateral DLPFC, the primary motor cortex (M1), and the left motor cortex, were selected, while quantitative assessment of the changes between the stimulation locations was limited due to the small number of included studies.

The roles of the M1 and DLPFC in pain modulation have long been established. A previous meta-analysis of high-frequency rTMS of M1 for neuropathic pain calculated effect sizes corresponding to a pain reduction of 12% and 13.7% on a visual analog scale [49, 50], and the analgesic effects were shown to be associated with changes in intracortical modulation, which depends on both the GABAergic and glutamatergic pathways [51–53]. On the other hand, a quantitative synthesis suggested that high-frequency rTMS over the DLPFC, an area of the cortex involved in pain perception and mood, should be considered as an alternative target in the management of neuropathic pain [29]; the mechanism was noted to be due to its connections with the limbic system and brainstem structures involved in descending modulation [54]. Moreover, several functional neuroimaging studies in humans have confirmed that, like M1 stimulation, rTMS of the DLPFC induces changes in the activity of a network of structures involved in the integration and modulation of pain signals, including the thalamus, brainstem, insular, and cingulate cortices [55–58]. Similar to our results, a recent study by Gatzinsky et al. also reported relief of persistent pain after DLPFC magnetic stimulation and at a 1-week follow-up, compared to baseline [59].

Nevertheless, we evaluated the 4- to 24-week follow-up effects of rTMS and found no significant pain reduction at either the mid- or long-term follow-up, which is not entirely consistent with Mhalla et al.'s opinion that the analgesic effects of repeated daily stimulations could last for 2-3 weeks after the last stimulation [60]. Current evidence indicates that the magnitude of diffuse analgesic effects induced by rTMS of the M1 and DLPFC, which can last several days after a single stimulation session and are reinforced by the repetition of sessions, depends both on the stimulation parameters (frequency, intensity, and pattern) and orientation of the coil [61]. The number of pulses per session in these included studies was lower than that in most previous studies (600-2000 pulses vs. 1000-2000 pulses) [62, 63], and the total number of pulses per treatment was also relatively small compared with other studies (6000-10,000 pulses vs. 10,000-20,000 pulses) [64]. Moreover, the average intervention duration was 7-14 days, and the negative results may be associated with the overall short treatment durations (average of 9.5 days with 3-10 treatment sessions). These treatment durations would be considered short relative to psychological or behavioral therapies for chronic pain [65].

The effects of rTMS were associated with some potential physiological mechanisms, as rTMS over the DLPFC could decrease amygdala activation-threatening stimuli [47]. The results showed that rTMS could generate a significant and sustained improvement in postconcussive symptoms in mild TBI, which is in accordance with the findings of Baeken et al. [66]. Moreover, rTMS has some superiority in that it can directly influence the brain, including regulating the prefrontal cortex, amygdala, and hippocampus [67], as well as expanding the cerebral blood vessels, thus improving microcirculation and cerebral blood flow [68]. As a result, the recovery of nerve function could be promoted, which could guide magnetoencephalogram activities to be normal and well organized [69].

Significant improvement in depression was observed only when TBI patients received 1 Hz right DLPFC rTMS. However, Cao et al. demonstrated that both high-frequency rTMS over the left DLPFC and low-frequency rTMS over the right DLPFC have similar therapeutic efficacy for the treatment of patients with major depressive disorder [70]. In addition, positive effects of rTMS on depression were confirmed by some clinical controlled studies and expert consensus [71], and the optimal stimulation dose was recommended as double 900 pulses on the right side or double 1500 pulses on the left side to maximize the antidepressant effect [4]. Although strong evidence suggests the effectiveness of rTMS on refractory depression, few studies have explored whether rTMS improves depression in TBI patients. A controlled study performed by Hoy and colleagues did not find an improvement in post-TBI depression [38]. To enhance the implication of rTMS on TBI patients with depression, unilateral or bilateral rTMS approaches need to be further investigated.

Moreover, the insignificance in cognition could be explained by the complexity of cognition modulation. The trials excluded patients with cognitive disorders, and the baseline cognitive function of patients in the included studies was normal, which represents an obstacle to detecting significant but small changes in cognition tests. Therefore, in light of the limitations above, some improvements in cognition following active treatment are encouraging, and well-designed RCTs with larger sample sizes should be conducted to determine the effect of rTMS on cognition.

### 4.1. Clinical Implications and Limitations

There are some strengths in this study. We presented the latest evidence-based quantitative review of the potential effects of rTMS on neuropathic pain and neuropsychiatric symptoms. The reviewed studies showed that rTMS can be conducted safely without major adverse events in TBI patients aged from 14 to 65 years old. This is a noteworthy result since it enables future research in the field, such as the exploration of unilateral or bilateral DLPF-rTMS techniques, which may have distinct effectiveness for ameliorating neuropathic pain, depression, and postconcussion symptoms but appear to have questionable efficacy for cognition in this population.

As shown in the funnel plot, there was some chance of publication bias. Furthermore, we cannot exclude the possibility of bias in our meta-analysis because of the small sample sizes and short intervention periods of the included studies. The length of follow-up in the included RCTs ranged from 1 to 24 weeks, and it is unknown whether the changes would persist beyond this time point. There were 5 studies showing a low risk of bias, while not all included RCTs generated an adequately randomized sequence or used a blinded method for the outcome measurement. The high risk of bias of the included studies may influence the reliability of the results. Due to the lack of included studies, it is difficult to execute further analysis on the effect of different timings and protocols of rTMS on TBI patients. Moreover, there is no evidence to support the efficacy and safety of rTMS for symptom improvement in children with TBI who are younger than 14 years old. The heterogeneity of outcome measures also limits the clinical implications.

## 5. Conclusions

Due to the small sample sizes and a lack of methodological quality, we can only make a fair recommendation that rTMS is a safe and effective tool to improve pain and postconcussive symptoms after TBI. More strict evaluation standards and high-quality RCT designs are necessary to further explore the effects of rTMS on TBI.

## Figures and Tables

**Table 1:** Characteristics of included studies.

Author, year	No. of participants (% men)	Age (y), range/mean (SD)	Duration since TBI/concussion (yrs)	Severity of TBI (mild/moderate/severe/unconfirmed)	Medications used	Outcome measures	Time points
Stilling et al., 2020	20 TBI with persistent PTH and PPCS (10%)	18-65; overall: 36.0 (11.4); G1: 40.3 (11.2); G2: 31.6 (10.4)	G1: 2.4(1.2); G2: 3.0(1.0)	G1: 10/0/0/0; G2: 10/0/0/0	OnabotulinumtoxinA: G1: 3, G2: 7; preventative headache medication: (1) Amitriptyline: G1: 3; G2: 1 (2) Topiramate: G1: 1; G2: 1 (3) Duloxetine: G1: 2; G2: 0 (4) Venlafaxine: G1: 1; G2: 0	(1) Headache: NRS (2) Cognition: MoCA, BCPSI, RPSQ (3) Function: HIT-6 (4) Depression: PHQ-9 (5) Anxiety: GAD-7 (6) Posttraumatic stress disorder: PTSD, PCL-5 (7) Quality of life: QOLIBRI	Baseline; midtreatment; posttreatment; 4 weeks/12 weeks/24 weeks posttreatment
Rao et al., 2019	30 TBI and anxiety G1: 13; G2:17 Men: 53.3%	Overall: 40 (14.4); G1: 40.2 (14.6); G2: 39.8 (14.2)	Not mentioned	G1: 15/2/0/0 G2: 13/0/0/0	Not mentioned	(1) Depression: HRSD (2) Clinical global impression-severity (CGI-S) scale (3) Clinical global impression-improvement (CGI-I) scale (4) The Beck scale for suicide ideation (BSSI) (5) Cognition: MoCA, RPSQ, BCPSI	Baseline; posttreatment; 4 weeks/8 weeks/12 weeks posttreatment
Moussavi et al., 2019	18 mild TBI; G1: 9; G2: 9; men: 50%	49.5 (12.4)	G1: <1.0; G2: >1.2	Not mentioned	Lamictal, zeldox, Zoloft, clonazapam, trazadone, amitriptyline, amitriptyline	(1) Symptom: RPSQ (2) Depression: MADRS	Baseline; posttreatment; 4 weeks/12 weeks posttreatment
Neville et al., 2019	30 TBI with chronic DAI; G1: 17; G2: 13; men: 90%	18-60; G1: 29.0 (10.35); G2: 32.62 (12.81)	>1.0	Not mentioned	No plans to change during the 90-day study period	(1) Cognition: TMT, COWAT, Stroop test, FPT, DST, SDT (2) Memory: HVLT and BVMT (3) Motor function: GPT	Baseline; posttreatment; 12 weeks posttreatment
Hoy et al., 2019	21 closed TBI; G1: 11; G2: 10; men: 47.6%	25-78; 46.29 (12.65)	Not mentioned	G1: 7/2/2/0; G2: 5/2/2/1	Antidepressant medication (yes/no): G1: 10/1; G2: 5/5; Mood stabiliser medication (yes/no): G1: 1/10; G2: 0/10; Benzodiazepine medication (yes regular/yes as needed/no): G1: 0/2/9; G2: 1/1/8; Antipsychotic medication (yes/no): G1: 1/10; G2:1/9;	(1) Depression: MADRS, IDS-CR, IDS-SR (3) Cognition: DST, TMT, arithmetic, RVALT, BVSMT, verbal fluency, Stroop test	Baseline; midtreatment; posttreatment
Siddiqi et al., 2019	15 mild TBI; G1: 9; G2: 5; men: 73.3%	G1: 43.0 (13.0); G2: 50.0 (18.0)	G1: 8.4 (8.2); G2: 8.1 (11.3)	Not mentioned	Not mentioned	(1) Depression: MADRS, DSM-5 (2) Personality: TCI, EB-SRMS, CB-CT, SRHLS, HIT-6	Baseline; midtreatment; posttreatment; 1 week/12 weeks/24 weeks posttreatment
Choi, et al., 2018	12 consecutive patients with mild TBI G1: 6; G2:6 Men: 50%	30-56; overall: 42.6 (8.7) G1: 43.2 (9.7) G2: 42 (8.4)	G1: 17.0 (7.5) G2: 14.3 (7.2)	G1: 6/0/0/0 G2: 6/0/0/0	Not mentioned	(1) Central pain: NPRS (2) Life quality: SF-36	Baseline; midtreatment; posttreatment; 1 week/2 weeks/4 weeks posttreatment
Lee et al., 2018	13 TBI; G1: 7; G2: 6; men: 69.2%	G1: 42.4 (11.3); G2: 41.3 (11.0)	G1: 3.9 (1.7); G2: 3.9 (1.9)	Not mentioned	Not mentioned	(1) Depression: MADRS (2) Cognition: TMT, SCWT	Baseline; posttreatment
Leung et al., 2018	29 mild TBI; G1: 14; G2: 15; men: 79.3%	G1: 33.0 (8.0); G2: 35.0 (8.0)	G1: 7.9 (6.9); G2: 8.3 (4.8)	Not mentioned	Maintain their existing medications	(1) Attention: CPT-II (2) Headache: NPRS, BPI (3) Cognition: WAIS-IV, Stroop test (4) Verbal: HVLT (5) Depression: HRSD (6) PTSD: CAPS	Baseline; 1 week/4 weeks posttreatment
Leung et al., 2016	24 mild TBI; G1: 12; G2: 12; men: 91.7%	G1: 41.2 (14.0); G2: 41.4 (11.6)	G1: 14.8 (14.7); G2: 13.6 (11.8)	Not mentioned	Medications	(1) Headache: NPRS (2) Attention: CPT-II (3) Depression: HRSD (4) PTSD: M-PTSD (5) Pain: BPI	Baseline; 1 week/4 weeks posttreatment
Franke et al., 2022	28 mild-to-moderate TBI; men: 85.7; G1 (active first): 13/14; G2 (sham first): 11/14	Overall: 45.6 (10.1); G1: 45.1 (11.3); G2: 46.0 (9.0)	Overall: 12.04 (6.8); G1: 11.43 (3.5); G2: 12.64 (9.1)	Mild or moderate	Not mentioned	(1) Depression: CAPS; PHQ-9 (2) Pain: McGill pain questionnaire (3) EEG (4) GSE; PSQI; TBI-QOL	Baseline; posttreatment for first condition (active or sham); pretreatment for second condition; posttreatment for second condition; 2 weeks posttreatment
Rodrigues et al., 2020	36 TBI and anxiety symptoms; G1: 18; G2: 18; men: 88.6%	18-65; G1: 32.8 (13.3); G2: 31.6 (11.3)	Not mentioned	Not mentioned	Not mentioned	(1) STAI-state (2) BDI-I (3) EF index	Baseline; midtreatment; posttreatment; 0 weeks posttreatment; 3 months

BCPSI: British Columbia Postconcussion Symptom Inventory; BDI-II: Beck Depression Inventory-II; BI: Barthel Index; BPI: Brief Pain Inventory; BSSI: Beck Scale for Suicide Ideation; BVMT: Brief Visuospatial Memory Test; BVSMT: brief visual spatial memory test; CAPS: Clinician-Administered PTSD Scale; CB-CT: Cognitive Testing-Cognitive Battery; CGI-I/CGI-S: Clinical Global Improvement-Severity/Improvement Scale Score; CMCT: Central Motor Conduction Time; COWAT: Controlled Oral Word Association Test; CPT-II: Conner's Continuous Performance Test II; DAI: Diffuse Axonal Injury; DSM-5: Diagnostic and Statistical Manual of Mental Disorders; DST: Digit Span Test; EB-SRMS: Emotion Battery-Self-Report Mood Scale; EEG: Electroencephalogram; EF index: Executive Function Index; FMA: Fugl-Meyer Assessment; FPT: Five-Point Test; G1: TMS group; G2: sham group; GAD-7: Generalized Anxiety Disorder Scale-7; GPT: Grooved Pegboard Test; GSE: General Self-Efficacy Scale; HAM-D: Hamilton Rating Scale for Depression; HIT-6: Headache Impact Test 6; HRSA/HRSD: Hamilton Rating Scale for Anxiety/Depression; HVLT: Verbal Hopkins Verbal Learning Test; IDS-CR/IDS-SR: Inventory of Depressive Symptomatology-Clinician Rated Version/Self-Rated Version; M1: primary motor cortex; MADRS: Montgomery-Asberg Depression Rating Scale; MEP: Motor Evoked Potential; MoCA: Montreal Cognitive Assessment; M-PTSD: Mississippi Scale for PTSD; NIHSS: National Institutes of Health Stroke Scale; NPRS: numeric pain rating scale; PCL-5: PTSD Checklist for DSM-5; PCL-M: PTSD Checklist-Military Version; PHQ-9: Patient Health Questionnaire-9; PPCS: persistent postconcussion symptoms; PSQI: Pittsburgh Sleep Quality Index; PTH: Posttraumatic Headache; PTSD: Posttraumatic Stress Disorder; QOLIBRI: Quality of Life after Brain Injury Questionnaire; RPQ: Rivermead Post-Concussion Symptoms Questionnaire; RPSQ-3: Rivermead Post-Concussion Symptoms Questionnaire-3; RVALT: Rey Verbal Auditory Learning Test; SCID: Structured Clinical Interview for DSM-IV Axes I & II; SCWT: Stroop Color-Word Test; SDT: symbol digit test; SF-36: MOS 36-Item Short-Form Health Survey; SRHLS: Self-Report Headache Likert Scores; SSRIs: Selective Serotonin Reuptake Inhibitors; STAI: State-Trait Anxiety Inventory; TBI: traumatic brain injury; TBI-QOL: traumatic brain injury quality of life; TCI: Temperament and Character Inventory; TMS: transcranial magnetic stimulation; TMT: Trail Making Test; WAIS-IV: Wechsler Adult Intelligence Scale; WMFT: Wolf Motor Function Test.

**Table 2:** TMS treatment and control group interventions in the included parallel group trials.

Author, year	TMS treatment group intervention						Sham control group intervention	Study duration	Adverse events
	Coil	Target brain region	Intensity	Frequency	Protocol frequency (sessions∗period)	Stimulation pulse/train			
Stilling et al., 2020	F8	Left DLPFC	70% RMT	10 Hz	1 session/d × 10 d	600/10	A sham air-film coil; the same protocol as TMS group	2 weeks	rTMS group: mild aggravation of headache; scalp discomfort; toothache; dizziness
Rao et al., 2019	A focal double 70 mm air-cooled coil	Right DLPFC	110% RMT	1 Hz	1 sessions/w × 4 w	1200/4	An identically appearing coil that produces the same sound and is the same weight as the active coil, but has negligible magnetic field strength	4 weeks	rTMS group: Headache: 5 Dizziness: 1 Blurred vision: 1 Tiredness: 1 Discomfort: 1 Eye twitching: 1 Sleep problem: 1 Depression: 1 Anxiety: 1 Puffy face: 1 Sham group: Headache: 12 Dizziness: 2 Discomfort: 1 Face twitching: 2 Sleep problem:4 Depression:1 Anxiety:3
Moussavi et al., 2019	F8	Left DLPFC	100% RMT	20 Hz	5 sessions/w × 2 w + 3 sessions/w × 1 w	750/25	The same protocol as TMS group	3 weeks	None
Neville et al., 2019	F8	Left DLPFC	110% RMT	10 Hz	1 session/d × 10 d	2000/40	A similar shape, color, and sound coil as TMS coil	12 weeks	Frequency of mild adverse events rTMS group vs. sham group (70.6% vs. 46.2%)
Hoy et al., 2019	F8	Bilateral DLPFC(right and then to left)	110% RMT	Right: 1 Hz Left: 10 Hz	5 sessions/w × 4 w	Right: 900/1 Left: 1500/30	The same protocol as TMS group; coil angled at 45° off the head	4 weeks	None
Siddiqi et al., 2019	F8	Bilateral DLPFC (right and then to left)	120% RMT	Right: 1 Hz Left: 10 Hz	1 session/d × 20 d	Right: 1000/1 Left: 4000/5	An alpha sham coil	5 weeks	Transient twitching and discomfort in the facial muscles: 7 in rTMS group; Worsening headaches: 1 in rTMS group and 1 in sham group; Presyncopal episode: 1 in rTMS group
Choi et al., 2018	F8	M1	90% RMT	10 Hz	5 sessions/w × 2 w	1000/20	The same protocol as TMS group	2 weeks	None
Lee et al., 2018	F8	Right DLPFC	100% RMT	1 Hz	5 sessions/w × 2 w	2000/50	A same size and shape coil as TMS coil	2 weeks	None
Leung et al., 2018	F8	Left DLPFC	80% RMT	10 Hz	4 sessions/w × 1 w	2000/20	180° away from the scalp after the RMT and with the coil side facing the scalp shielded with a molded cover containing two layers of Giron magnetic shielding film	1 week	Not mentioned
Leung et al., 2016	F8	Left MC	80% RMT	10 Hz	3 sessions/w × 1 w	2000/20	Visualize the movement of coil and treatment beam over their own cortices on the monitor, and heard the sound and felt the vibration of the stimulation just like the patients receiving the active treatment	1 week	None
Franke et al., 2022	F8	Right DLPFC	80% RMT for day1 and 100% RMT thereafter	10 Hz	1 sessions/d × 5 d	—	Stimulation set at 25% RMT with the coil tilted 90 degrees from the scalp	5 days	Not mentioned

F8: figure of 8 coil; ABP: abductor pollicis brevis; RMT: resting motor threshold; DLPFC: dorsolateral prefrontal cortex; M1: primary motor cortex area, MT: motor threshold; MC: motor cortex.

**Table 3:** The Cochrane tool of assessing risk of bias for methodological assessment (RoB 2.0 tool).

Article, year	Randomization process	Deviations from intended interventions	Missing outcome data	Measurement of the outcome	Selection of the reported	Overall
Stilling et al., 2020	Low	Low	Low	Unclear	Low	Unclear
Moussavi et al., 2019	Low	High	Low	High	Low	High
Neville et al., 2019	Low	Low	Low	Unclear	Low	Unclear
Hoy et al., 2019	Low	Low	Low	Low	Low	Low
Siddiqi et al., 2019	Unclear	Low	Low	Low	Low	Unclear
Choi et al., 2018	Low	Low	Low	Low	Low	Low
Lee et al., 2018	Low	Low	Low	High	Low	High
Leung et al., 2018	Low	Low	Low	Low	Low	Low
Leung et al., 2016	Low	Low	Low	Low	Low	Low
Rao et al., 2019	Low	Low	Low	Low	Low	Low
Franke et al., 2022	Unclear	Low	Low	Low	Low	Unclear

RoB: risk of bias.

## Data Availability

The data used to support the findings of this study are available from the corresponding authors upon request.

## Supplementary Materials

Supplementary 1 search strategies for all databases shown in (Appendix S1).

Supplementary 2 summary of findings table: GRADE levels of evidence for studies of TMS (Appendix S2).
