# Supplementary material for: Repetitive Transcranial Magnetic Stimulation for Neuropathic Pain and Neuropsychiatric Symptoms in Traumatic Brain Injury: A Systematic Review and Meta-Analysis
Source: Neural Plast. 2022 Jul 30;2022:2036736. doi: 10.1155/2022/2036736 (PMC9357260; doi:10.1155/2022/2036736)
Supplement: Supplementary 1 — search strategies for all databases shown in (Appendix S1). [file 2036736.f1.doc]

**Search strategies for all databases**

1. Search Strategy for PubMed:

#1 “parallel”[Text Word] OR “controlled trial*”[Text Word] OR “random*”[Text Word] OR “randomi*”[Text Word] OR “intervention*”[Text Word] 2,629,584

#2 “clinical trial”[PT]

#3 #1 OR #2

#4 “review”[Title]

#5 #3 NOT #4

#6  “in rat” OR “animal*”[Title]

#7 #5 NOT #6

#8 "traumatic brain injury"[All Fields] OR "traumatic epidural hematoma"[All Fields] OR "traumatic subdural hematoma"[All Fields] OR "traumatic intracerebral haemorrhage"[All Fields] OR "brain damage*"[Title/Abstract] OR "brain trauma*"[Title/Abstract] OR "brain injury*"[Title/Abstract] OR "brain laceration*"[Title/Abstract] OR "head injury*"[Title/Abstract] OR "cerebral injury*"[Title/Abstract] OR "head trauma*"[Title/Abstract] OR"concussion"[Title/Abstract] OR "hematoma"[MeSH Terms] OR "traumatic stress disorder"[All Fields]

#9 "transcranial magnetic stimulation"[All Fields] OR "TMS"[All Fields] OR "neuromodulation*"[All Fields]

#11 #7 AND #8 AND #9

2. Search Strategy for EMBASE

(((parallel OR controlled) AND trial* OR 'random' OR randomi OR 'intervention') AND clinical AND trial OR randomi OR intervention OR random OR parallel) AND ('traumatic brain injury':ab,ti OR 'traumatic epidural hematoma':ab,ti OR 'traumatic subdural hematoma':ab,ti OR 'traumatic intracerebral haemorrhage':ab,ti OR 'brain damage':ab,ti OR 'brain trauma':ab,ti OR 'brain injury':ab,ti OR 'brain laceration':ab,ti OR 'head injury':ab,ti OR 'cerebral injury':ab,ti OR 'head trauma':ab,ti OR 'traumatic':ab,ti) AND ('transcranial magnetic stimulation' OR 'tms' OR 'neuromodulation')

3. Search Strategy for Cochrane Library

#1 parallel OR 'controlled trial*' OR 'random*' OR 'randomi*' OR 'intervention*'

#2 [publication type] “clinical trial”

#3 #1 AND #2

#4 'traumatic brain injury*' OR 'traumatic' OR 'brain injury*' OR 'brain damage*' OR 'brain trauma*' OR ' cerebral injury' OR 'TBI' OR 'brain insult'OR 'brain laceration*' OR 'laceration*，brain' OR 'brain injury*, focal' OR 'focal brain injury' OR 'injur*, focal brain' OR 'cerebral injury'

#5 ' transcranial magnetic stimulation ' OR ' TMS ' OR ' neuromodulation*'

#6 #3 And #4 And #5

4. Search Strategy for Web of Science

#1 TS=(brain injury* OR brain trauma* OR brain damage* OR cerebral damage* OR brain laceration*' OR 'brain concussions' OR 'cerebral contusion and laceration*' OR 'hematoma*' OR 'fracture of skull' OR ' traumatic brain injury* OR traumatic subdural hematoma OR cerebral injury* OR post-traumatic* )

#2 TS=(transcranial magnetic stimulation OR TMS OR neuromodulation)

#3 #1 AND #

Search in:All Databases

Collections:All

5. Search Strategy for CINAHL (Ebsco)

S1 ( TX("parallel " OR " controlled trials" OR " random " OR " randomi " OR " intervention ") ) OR TX ("clinical trial*") OR ( TI (controlled trial OR "clinical trial*") )

S2 TX("brain injury*" OR "brain trauma*" OR "brain damage*" OR "cerebral damage*" OR "brain laceration*" OR "brain concussions" OR "cerebral contusion and laceration*" OR "hematoma*" OR "fracture of skull" OR "TBI" OR "head trauma" OR " traumatic subdural hematoma " OR " traumatic brain injury ") OR " post-traumatic "

S3 AB(transcranial magnetic stimulation OR TMS OR neuromodulation")

S4 S1 AND S2 AND S3
