# Supplementary material for: Repetitive Transcranial Magnetic Stimulation for Neuropathic Pain and Neuropsychiatric Symptoms in Traumatic Brain Injury: A Systematic Review and Meta-Analysis
Source: Neural Plast. 2022 Jul 30;2022:2036736. doi: 10.1155/2022/2036736 (PMC9357260; doi:10.1155/2022/2036736)
Supplement: Supplementary 2 — summary of findings table: GRADE levels of evidence for studies of TMS (Appendix S2). [file 2036736.f2.docx]

**e Table | Summary of findings table-GRADE levels of evidence for studies of TMS.**

| **rTMS compared to other treatments with TBI** |
| --- |
| **Patient or population:** people with TBI or PTSD **Intervention:** rTMS **Comparison:** sham treatment |

| **Certainty assessment** | | | | | | | | **№ of patients** | | **Effect** | | **Certainty** |
| --- | --- | --- | --- | --- | --- | --- | --- | --- | --- | --- | --- | --- |
| **Outcomes** | **№ of studies** | **Study design** | **Risk of bias** | **Inconsistency** | **Indirectness** | **Imprecision** | **Other considerations** | **tms** | **sham** | **Relative (95% CI)** | **Absolute (95% CI)** |  |
| *Neuropathic Pain* | 4 | RCT | not serious | serious^2^ | not serious | serious^5^ | publication bias strongly suspected^4^ | 42 | 43 | - | MD 1.00 lower (1.76 lower to 0.25 lower) | ⨁◯◯◯ VERY LOW |
| *Depression (evaluated by MADRS)* | 5 | RCT | not serious | serious^2^ | not serious | serious^5^ | none | 53 | 47 | - | MD 2.53 lower (5.92 lower to 0.86 higher) | ⨁⨁◯◯ LOW |
| *Depression (evaluated by HRDS)* | 2 | RCT | not serious | not serious | not serious | serious^5^ | none | 27 | 32 | - | MD 1.03 lower (3.56 lower to 1.49 higher) | ⨁⨁⨁◯ MODERATE |
| *Depression (evaluated by PHQ-9)* | 1 | RCT | serious^6^ | serious^2^ | not serious | serious^5^ | none | 14 | 12 | - | MD 0.76 lower (1.78 lower to 0.26 higher) | ⨁◯◯◯ VERY LOW |
| Symptons *(evaluated by RPQ)* | 2 | RCT | not serious | not serious | not serious | serious^5^ | none | 19 | 19 | - | MD 0.29 lower (1.55 lower to 0.97 higher) | ⨁⨁⨁◯ MODERATE |
| *Cognition (evaluated by TMT)* | 3 | RCT | not serious | not serious | not serious | serious^5^ | none | 41 | 40 | - | MD 5.15 lower (20.19 lower to 9.89 higher) | ⨁⨁⨁◯ MODERATE |
| *Cognition (evaluated by SCWT)* | 2 | RCT | not serious | not serious | not serious | serious^5^ | none | 24 | 27 | - | MD 0.66 higher (6.52 lower to 7.84 higher) | ⨁⨁⨁◯ MODERATE |

GRADE: Grading of Recommendations, Assessment, Development and Evaluations; RCT: Randomized Controlled Trial; MADRS: Montgomery Asberg Depression Rating Scale; HRSD: Hamilton Rating Scale for Depression; PTSD: posttraumatic stress disorder; PCL: PTSD Checklist; RPQ: Rivermead Post-Concussion Questionnaire; TMT: Trail Making Test; SCWT: Stroop Color Word Test.
